# Barriers to the Access of Bevacizumab in Patients with Solid Tumors and the Potential Impact of Biosimilars: A Physician Survey

**DOI:** 10.3390/ph10010019

**Published:** 2017-01-28

**Authors:** Bradley J. Monk, Philip E. Lammers, Thomas Cartwright, Ira Jacobs

**Affiliations:** 1 Arizona Oncology (US Oncology Network), University of Arizona, College of Medicine, Creighton University School of Medicine at St. Joseph’s Hospital, 2222 E. Highland Ave., Suite 400, Phoenix, AZ 85016, USA; Bradley.Monk@usoncology.com; 2 Meharry Medical College, 1005 Dr. D.B. Todd Jr. Blvd., Nashville, TN 37208-3501, USA; plammers@mmc.edu; 3 Florida Cancer Affiliates, 433 SW 10th Street, Ocala, FL 34471, USA; thomas.cartwright@usoncology.com; 4 Pfizer Inc., 235 East 42nd Street, New York, NY 10017-5755, USA

**Keywords:** access to health care, bevacizumab, biosimilars, colorectal cancer, non–small-cell lung cancer, ovarian cancer

## Abstract

Access to bevacizumab, an important component of oncology treatment regimens, may be limited. This survey of oncologists in the US (*n* = 150), Europe (*n* = 230), and emerging markets (EM: Brazil, Mexico, and Turkey; *n* = 130) examined use of and barriers to accessing bevacizumab as treatment of advanced solid tumors. We also assessed the likelihood that physicians would prescribe a bevacizumab biosimilar, if available. Bevacizumab was frequently used as early-line therapy in metastatic colorectal cancer, metastatic non-squamous non–small-cell lung cancer, and metastatic ovarian cancer (all markets), and as a second-line therapy in glioblastoma multiforme (US, EM). A greater percentage of EM-based physicians cited access-related issues as a barrier to prescribing bevacizumab versus US and EU physicians. Lack of reimbursement and high out-of-pocket costs were cited as predominant barriers to prescribing and common reasons for reducing the number of planned cycles. Overall, ~50% of physicians reported they “definitely” or “probably” would prescribe a bevacizumab biosimilar, if available. Efficacy and safety data in specific tumor types and lower cost were factors cited that would increase likelihood to prescribe a bevacizumab biosimilar. A lower cost bevacizumab biosimilar could address the unmet needs of patients and physicians worldwide, and may have the greatest impact on patient outcomes in EM.

## 1. Introduction

Bevacizumab (Avastin^®^; Genentech Inc., South San Francisco, CA, USA, and Roche Registration LTD, Welwyn Garden City, UK) is a recombinant humanized immunoglobulin G1 monoclonal antibody targeting vascular endothelial growth factor (VEGF) [1,2,3], an endothelial cell-specific mitogen and proangiogenic protein [4]. Binding of bevacizumab prevents the interaction of VEGF with its receptors VEGFR-1 (Flt-1) and VEGFR-2 (KDR), and disrupts the formation of new tumor vasculature [2,5,6]. Bevacizumab can, therefore, inhibit tumor growth [2,5,6] and is used to treat a variety of human cancers. In the United States (US) and European Union (EU) bevacizumab is indicated for the treatment of patients with metastatic colorectal cancer (mCRC), advanced or metastatic non-squamous non–small-cell lung cancer (mNSCLC), metastatic renal cell carcinoma, and certain gynecologic malignancies [1,3]. Additionally, it is indicated for the treatment of patients with glioblastoma (GBM) in the US [1] and patients with metastatic breast cancer (mBC) in the EU [3]. 

Randomized phase III trials demonstrate that adding bevacizumab to standard chemotherapy prolongs progression-free and/or overall survival in patients with mCRC, advanced non-squamous NSCLC, metastatic ovarian cancer (mOC), cervical cancer, and mBC [7,8,9,10,11]. Bevacizumab alone, or in combination with chemotherapy, has also shown clinical activity in patients with recurrent GBM [12]. Thus, bevacizumab is an important component of oncology treatment regimens. However, access to this biologic may be limited, similar to what has been demonstrated for other biologic therapies [13,14,15]. Other angiogenesis inhibitors, such as ramucirumab and aflibercept, demonstrate similar efficacy and safety, but may not offer economic advantages over bevacizumab [16]. This is relevant considering access issues related to insurance coverage and treatment costs create barriers to the use of biologics in many countries [13,14,15]. 

Biologics, including bevacizumab, are large, complex proteins that are produced in living cells [17,18,19]. Biosimilars are biologic products with demonstrated similarity to a licensed biologic (i.e., reference or originator) and have no clinically meaningful differences in product safety, purity, or potency [20,21,22]. However, a biosimilar is not an exact copy of its originator as the manufacturing processes used for biosimilar development likely differ from those of the originator [17,19]. This stands in contrast to traditional small-molecule drugs, which have simple structures that are readily defined and can be exactly replicated using controlled and predictable chemical reactions to produce a generic equivalent [17,19].

The development and introduction of lower cost biosimilars may provide an opportunity to increase patient access to biologic therapies and has the potential to generate savings and efficiencies for healthcare systems. Patents for bevacizumab will soon expire in Europe and the US [23], and several bevacizumab biosimilars are in development [24]. Accordingly, the current study examined access to bevacizumab and potential barriers to its use in the US, EU, and emerging markets (EM). This study also evaluated the likelihood that physicians would prescribe a bevacizumab biosimilar, if one was available.

## 2. Results

### 2.1. Patient Load and Cancer Patient Population

Across individual countries, patient load tended to be higher for mBC (*n* = 13–119) and lower for GBM (*n* = 6–18) versus mCRC (*n* = 18–58), mNSCLC (*n* = 15–60), and mOC (*n* = 9–39) (Supplementary Table S1). In the US, patients with solid tumors were most likely to have private insurance (44%) versus supplementary coinsurance (22%), government-funded insurance (27%), no insurance/out-of-pocket (6%), and other (1%). Across individual countries in the EU and EM, respectively, patients were most likely to receive government-funded insurance (66%–89% and 39%–59%) versus private insurance (4%–18% and 23%–39%), supplementary coinsurance (2%–14% and 6%–15%), no insurance/out-of-pocket (1%–4% and 6%–19%), and other (1%–4% and 0%–2%).

### 2.2. Commonly Followed Treatment Guidelines

**Figure 1:**
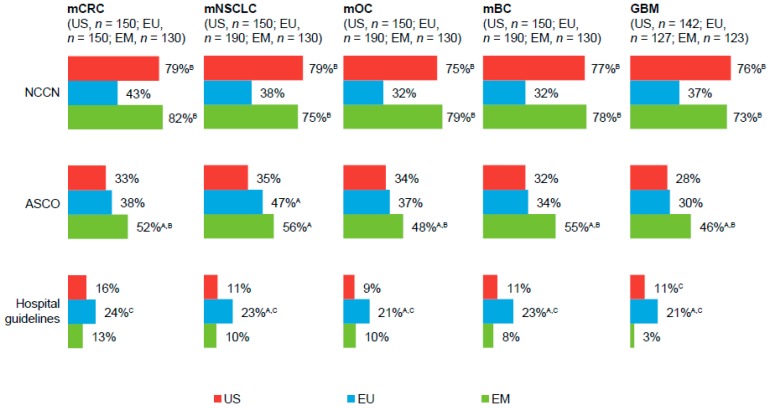
Treatment guidelines followed by physicians, by primary tumor type ^a^. ^A,B,C^ Letters indicate a significant difference between subgroups (*p* < 0.05): A = US; B = EU; C = EM. ^a^ Percentages are based on respondents who reported treating patients who had each primary tumor type. EU, European Union (United Kingdom (UK), Italy, Germany, and France); EM, emerging markets (Brazil, Mexico, and Turkey); mCRC, metastatic colorectal cancer; NCCN, National Comprehensive Cancer Network; ASCO, American Society of Clinical Oncology; mNSCLC, metastatic non-squamous non–small-cell lung cancer; mOC, metastatic ovarian cancer; mBC, metastatic breast cancer; GBM, glioblastoma.

Physicians in all regions reported following National Comprehensive Cancer Network, American Society of Clinical Oncology, and, to some extent, hospital guidelines when treating patients with solid tumors (Figure 1; Supplementary Table S2). In the EU and EM, physicians also followed European Society of Medical Oncology (ESMO) and National/Ministry of Health guidelines (Supplementary Table S2).

### 2.3. Bevacizumab Treatment

A majority of physicians in the US, EU, and EM, respectively, reported “always” or “frequently” prescribing bevacizumab as first-line therapy in the treatment of Kirsten rat sarcoma viral oncogene homolog (*KRAS*)/*RAS* mutant (Mut) or wild-type (WT)/unknown mCRC (85%–91%, 61%–87%, and 66%–87%), epidermal growth factor receptor (*EGFR*) Mut or WT or unknown/not tested mNSCLC (59%–84%, 38%–55%, and 46%–65%), and mOC (58%, 69%, and 46%). Most physicians in the US, EU, and EM, respectively, also reported “always” or “frequently” prescribing bevacizumab as maintenance therapy (mCRC: 79%, 68%, 49%; mNSCLC: 67%–74%, 47%–48%, 43%–57%) or second-line therapy (mCRC: 73%, 63%, 41%; mNSCLC: 47%–51%, 25%–32%, 24%; platinum-sensitive or resistant mOC: 58%–62%, 34%–50%, 33%–40%). 

Overall, bevacizumab prescribing for the treatment of patients with mBC was low; <50% of physicians in any region reported “always” or “frequently” prescribing bevacizumab as first-line, maintenance, or second-line therapy, and nearly 40% of US physicians reported “never” prescribing bevacizumab as any line of therapy. Fewer than 50% of physicians in any region reported “always” or “frequently” prescribing bevacizumab as first-line therapy for the treatment of patients with GBM; however, 79% of US and 61% of EM physicians reported “always” or “frequently” prescribing bevacizumab as second-line therapy versus 42% of EU physicians.

### 2.4. Access to Bevacizumab and Barriers to Treatment

Of respondents who do not frequently prescribe bevacizumab (Supplementary Figure S1), a greater percentage of physicians from the EM (35%–82%) versus the US (10%–43%) and EU (16%–41%) reported access as a barrier to more-frequent bevacizumab prescribing across all tumor types and treatment settings. However, by tumor type, approximately 40% of US physicians cited access as a concern for the treatment of patients with mCRC in the first-line setting; approximately 30% considered access as a barrier to frequent bevacizumab prescribing in first-line mBC. 

Ease of access to bevacizumab was measured using a seven-point scale in which a score of 1 = not at all easy and 7 = very easy. Physicians in the US and EU found bevacizumab easier to access (significantly higher mean response) versus physicians in the EM, respectively, for treatment of patients with mCRC (5.64, 5.68, and 4.88), mNSCLC (5.35, 4.99, and 3.88), and mOC (4.67, 5.19, and 3.99). Physicians in the US found bevacizumab easier to access versus those in the EU or EM, respectively, when treating GBM (5.11, 4.22, and 3.86). In contrast, EU physicians found bevacizumab easier to access versus US and EM physicians when treating mBC (3.53, 5.01, and 3.46, US, EU, and EM, respectively).

**Figure 2:**
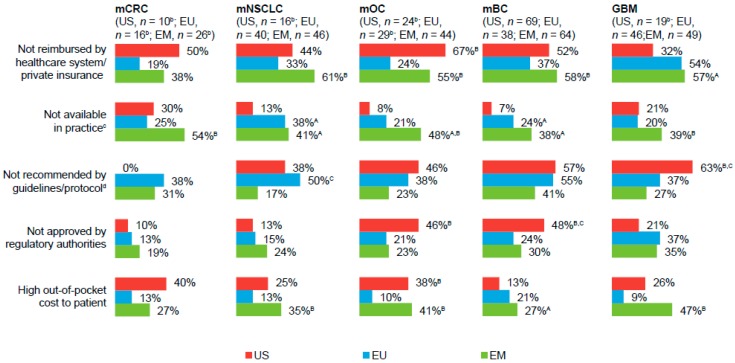
Barriers to bevacizumab access, by primary tumor type ^a^. ^A,B,C^ Letters indicate a significant difference between subgroups (*p* < 0.05): A = US; B = EU; C = EM. ^a^ Percentages are based on respondents who considered access to bevacizumab as difficult (a score ≤3 on a scale from 1 = not at all easy to 7 = very easy). ^b^ Small sample size; results interpreted with caution. ^c^ Answer option “not available in practice” indicates that physicians did not have the drug on hand. ^d^ Answer option “not recommended by guidelines/protocol” indicates there was no specific recommendation for bevacizumab use. EU, European Union (UK, Italy, Germany, and France); EM, emerging markets (Brazil, Mexico, and Turkey); mCRC, metastatic colorectal cancer; mNSCLC, metastatic non-squamous non–small-cell lung cancer; mOC, metastatic ovarian cancer; mBC, metastatic breast cancer; GBM, glioblastoma.

Of physicians who considered access to bevacizumab as difficult (a score ≤3 on a seven-point scale; Figure 2), “not reimbursed by healthcare system/private insurance” and/or “high out-of-pocket costs to the patient” were cited as predominant access-related barriers to prescribing bevacizumab, particularly among EM-based physicians. “Not available in practice” (EU- and EM-based physicians) and “not recommended by guidelines/protocol” (US- and EU-based physicians) were other common barriers to bevacizumab access.

Across tumor types, a greater percentage of EM-based physicians reported “always” or “very often” having to provide medical justification for prescribing bevacizumab due to cost of treatment issues (US, 14%–33%; EU, 11%–25%; EM, 33%–38%). However, one-third of US physicians reported “always” or “very often” having to provide medical justification for bevacizumab use for treatment of patients with mBC. Regardless of tumor type, physicians in the EU were least likely to reduce the number of planned bevacizumab cycles due to access-related issues, with the percentages of physicians who reported “frequently” or “occasionally” having to reduce the number of cycles as follows: US, 32%–56%; EU, 17%–27%; and EM, 35%–50%.

**Figure 3:**
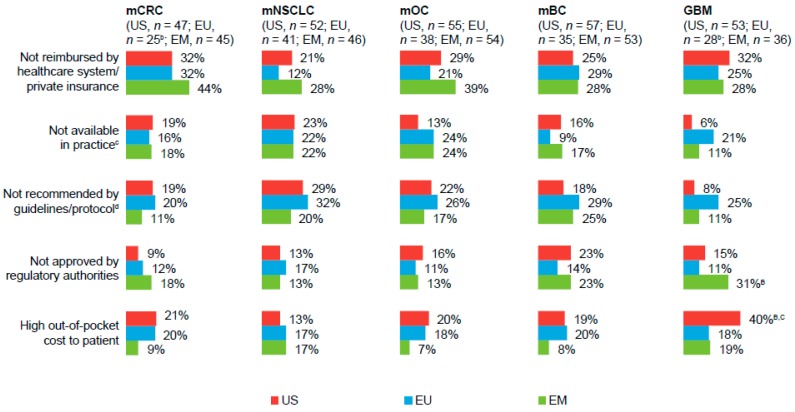
Reasons for reducing the number of planned bevacizumab cycles, by primary tumor type ^a^. ^A,B,C^ Letters indicate a significant difference between subgroups (*p* < 0.05): A = US; B = EU; C = EM. ^a^ Values represent the percentages of respondents who reported “frequently” or “occasionally” (a score of 3 or 4 on a scale from 1 = never to 4 = frequently) having to reduce the number of planned bevacizumab cycles among those who reported treating each primary tumor type. ^b^ Small sample size; results interpreted with caution. ^c^ Answer option “not available in practice” indicates that physicians did not have the drug on hand. ^d^ Answer option “not recommended by guidelines/protocol” indicates there was no specific recommendation for bevacizumab use. EU, European Union (UK, Italy, Germany, and France); EM, emerging markets (Brazil, Mexico, and Turkey); mCRC, metastatic colorectal cancer; mNSCLC, metastatic non-squamous non–small-cell lung cancer; mOC, metastatic ovarian cancer; mBC, metastatic breast cancer; GBM, glioblastoma.

Among physicians who ever had to reduce the number of bevacizumab cycles, “not reimbursed by healthcare system/private insurance” was cited as a predominant reason for limiting the number of cycles, across all markets and tumor types (Figure 3). A greater percentage of physicians in the US versus those in the EU and the EM also cited “high out-of-pocket costs to the patient” as a reason for limiting the number of bevacizumab cycles in the treatment of patients with GBM. Conversely, a greater percentage of physicians in the EM, versus those in the US or EU, had to reduce the number of bevacizumab cycles for treatment of patients with GBM because its use was not approved by regulatory authorities. Across all markets and tumor types, roughly one-half of physicians reported that reducing the number of bevacizumab cycles would “likely” or “very likely” impact patient quality of life and survival.

### 2.5. Reactions to a Biosimilar to Bevacizumab

**Figure 4:**
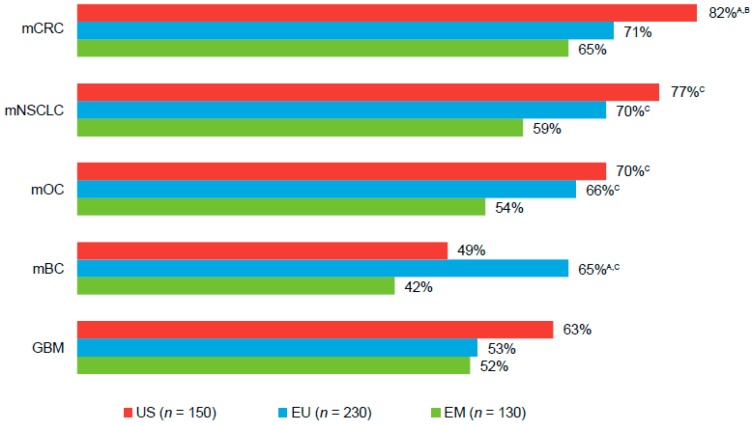
Likelihood a physician would prescribe a bevacizumab biosimilar, by primary tumor type ^a^. ^A,B,C^ Letters indicate a significant difference between subgroups (*p* < 0.05): A = US; B = EU; C = EM. ^a^ Values represent the percentages of respondents who indicated they “definitely” or “probably” (a score of 4 or 5 on a scale from 1 = definitely would not prescribe to 5 = definitely would prescribe) would prescribe biosimilar bevacizumab among those who reported treating each primary tumor type. EU, European Union (UK, Italy, Germany, and France); EM, emerging markets (Brazil, Mexico, and Turkey); mCRC, metastatic colorectal cancer; mNSCLC, metastatic non-squamous non–small-cell lung cancer; mOC, metastatic ovarian cancer; mBC, metastatic breast cancer; GBM, glioblastoma.

Across all tumor types and markets, 42%–82% of physicians reported they “definitely” or “probably” would prescribe a bevacizumab biosimilar, if available (Figure 4). Physicians in the US and EU expressed particular interest in a bevacizumab biosimilar for the treatment of patients with mCRC, mNSCLC, or mOC. Across tumor types, physicians in all regions cited efficacy and cost as key drivers for prescribing a bevacizumab biosimilar, if available (efficacy: 41%–51%, 41%–47%, and 26%–43%; cost: 33%–44%, 45%–48%, and 29%–35%, US, EU, and EM, respectively). Physicians also cited efficacy and lack of clinical trial data as their rationale against prescribing a bevacizumab biosimilar (efficacy: 22%–50%, 0%–18%, and 14%–32%; lack of clinical trial data: 0%–15%, 15%–28%, and 16%–34%, US, EU, and EM, respectively).

Among those who “probably” or “definitely” would not prescribe a bevacizumab biosimilar, efficacy and safety data in specific tumor types and a larger cost reduction than what was estimated (i.e., 20% discount vs originator) for this study were cited as factors that would increase the likelihood to prescribe a bevacizumab biosimilar for US, EU, and EM patients, respectively (efficacy data: 64%–74%, 56%–70%, and 67%–80%; safety data: 48%–59%, 35%–44%, and 53%–64%; lower cost: 44%–86%, 46%–79%, and 37%–87%). Across markets, 40%–46% of physicians ranked *KRAS/RAS* Mut mCRC/first-line as the “number 1” tumor type and treatment setting in which a bevacizumab biosimilar would have the greatest impact on patient outcomes among patients with solid tumors. This was followed by *KRAS/RAS* WT/unknown mCRC/first-line (10%–17% of physicians), *EGFR* WT or unknown/not tested mNSCLC/first-line (8%–13%), and mOC/first-line (3%–10%).

## 3. Discussion

This study demonstrates that access concerns create barriers to bevacizumab treatment in patients worldwide. Overall, access was not a primary barrier to frequent bevacizumab prescribing among US and EU physicians; however, access-related issues were cited as a concern among a small base of US physicians for treatment in the first-line setting for patients with mCRC and mBC. 

Across tumor types and markets, access issues related to lack of reimbursement to and/or high out-of-pocket costs for the patient were cited as predominant barriers to bevacizumab use. In the EM, where access concerns were considered a primary barrier to frequency of bevacizumab prescribing and where overall access to bevacizumab was difficult, physicians frequently had to provide medical justification for bevacizumab use due to issues related to treatment costs. This may be related to the fact that patients from the EM were more likely than patients from the US or EU to pay for medical expenses out-of-pocket. Issues related to lack of reimbursement and/or high out-of-pocket costs to the patient were also cited as predominant reasons for reducing the number of planned bevacizumab cycles; a change in treatment that, for physicians in all three markets, was considered to impact patient quality of life and survival. 

These findings are consistent with other studies demonstrating barriers to the use of biologic therapies [13,14,15]. Data from the ESMO European Consortium on the availability of antineoplastic agents show disparities in access to bevacizumab and other targeted therapies in Europe [14], with some Western (e.g., Greece, Turkey, and the UK) and many Eastern European (e.g., Armenia, Romania, and Russia) countries reporting only occasional access to bevacizumab or access to bevacizumab only half of the time for treatment of patients with lung cancer, mBC, CRC, or ovarian cancer. Issues related to cost to patients and insurance coverage have been reported as common barriers to the use of rituximab in patients with hematologic malignancies and for trastuzumab in patients with human epidermal growth factor receptor 2–amplified breast cancer, particularly among several EM countries (e.g., Brazil, Mexico, Turkey, and Russia) [13,15]. These issues also led to changes in treatment strategy (e.g., cancel, delay, or reduction in planned cycles) with rituximab or trastuzumab [13,15], which is consistent with the present findings for bevacizumab. The ESMO European Consortium study also demonstrates disparities in treatment cost for bevacizumab across Europe [14], which may contribute to its restricted accessibility in some countries. This is especially relevant in countries where patients are responsible for the full cost of treatment with bevacizumab [14] and where cancer drugs, in general, are less affordable (e.g., China and India) [25].

Barriers to bevacizumab access involved more than issues related to treatment costs. Across tumor types, physicians in the EU and EM commonly cited “not available in practice” and physicians in the US and EU commonly cited “not recommended by guidelines/protocol” as barriers to bevacizumab use. Problems related to manufacturing and distribution have been cited as a barrier to accessibility of anticancer medicines in Europe, and may contribute to the limited availability of bevacizumab in EM countries. Apart from access-related issues, physicians also cited “patient clinical factors,” “bevacizumab not the optimal treatment option in this tumor type,” and “not convinced of bevacizumab’s efficacy in this setting” as reasons for not frequently prescribing bevacizumab.

The current findings also suggest that bevacizumab use would increase across all markets and tumor types if a lower-cost biosimilar version with demonstrated efficacy similar to the originator bevacizumab was available. However, availability of clinical trial efficacy and safety data in specific tumor types and greater cost reductions will play an important role in the likelihood of physicians to prescribe a bevacizumab biosimilar. In general, the lower cost is likely to offer an advantage to prescribing the bevacizumab biosimilar over the originator or other angiogenesis inhibitors that demonstrate similar efficacy and safety, but may not offer any economic advantages over bevacizumab [16]. Moreover, cost reduction may have the largest impact on prescribing a bevacizumab biosimilar in the EM where, as noted above, patients are more likely to pay for medical expenses out-of-pocket and physicians must frequently provide justification for and experience greater difficulty obtaining bevacizumab due to cost of treatment issues. Finally, since physician decisions to prescribe bevacizumab were likely influenced by economic factors, the anticipated lower cost of biosimilars may lead to increased bevacizumab biosimilar use.

Although physicians in the US and EU did not consider cost as a primary barrier to bevacizumab access, their interest in the biosimilar version was quite high, especially for the tumor types and treatment settings in which bevacizumab is frequently used, such as early-line therapy in mCRC, mNSCLC, and mOC. Across markets, physicians suggested that a bevacizumab biosimilar would have the greatest impact on patient outcomes among patients with mCRC and mNSCLC treated in the first-line setting. This is consistent with current approved indications for and recommended use of bevacizumab in patients with cancer [1,3,26,27,28,29,30,31]. 

More than half of US and EU physicians reported always or frequently prescribing bevacizumab as first-line therapy for treatment of patients with mOC, and 3%–10% rated this as the “number 1” tumor type and treatment setting in which a bevacizumab biosimilar would have the greatest impact on patient outcomes among patients with solid tumors. The impact of a bevacizumab biosimilar on patient outcomes among women with cervical cancer should also be considered. Bevacizumab in combination with doublet chemotherapy provides a survival benefit over chemotherapy alone in patients with metastatic, persistent, or recurrent cervical cancer [11] and this triplet regimen is now approved in the US, EU, and other countries worldwide for the treatment of patients with advanced cervical cancer [1,3,32]. Bevacizumab treatment in cervical cancer was not assessed in the current study; however, its use in patients with this disease is likely to increase. Availability of a bevacizumab biosimilar has the potential to improve patient outcomes among women with cervical cancer, particularly for those who live in less-developed regions, where this disease accounts for almost 12% of all cancers in women (vs ~3% in more-developed regions) [33].

As with all survey research, interpretation of these results may be limited by the accuracy of the physicians’ reporting of events and generalizability of our findings to physicians from other countries that were not examined in this study. Other limitations of the current study include the format in which responses were collected and small sample sizes. For instance, survey questions with answers that are framed in the multiple-choice format or that employ a rating scale may fail to capture all details of a physician’s experience or other possible reasons for having difficulty with obtaining bevacizumab for his or her patients.

Overall, these data demonstrate that access-related issues create a barrier to bevacizumab use worldwide, but particularly among physicians in the EM. Access-related issues also led to changes in treatment strategy (i.e., reduction in planned cycles) that are considered by physicians to impact patient quality of life and survival. The availability of other biosimilars in Europe has increased the use of some biologic drugs, providing evidence for improved patient access to treatment. In the United Kingdom (UK), overall use of granulocyte colony-stimulating factor increased by 13% in the first year after and a further 17% in the second year following the introduction of biosimilar filgrastim [34]. In addition, a study calculated that the potential cost savings generated by switching 10,000 patients in Germany, France, Italy, Spain, and the UK from originator to biosimilar filgrastim could allow for up to an additional 347–1213 patients to be treated with rituximab or 132–461 patients to receive trastuzumab [35]. A bevacizumab biosimilar could offer the same opportunity to address the needs of patients with cancer by increasing access to bevacizumab, and generating cost savings that could be redistributed to expand patient access to other biologic therapies. Furthermore, our results suggest that a bevacizumab biosimilar may have the greatest impact on patients’ outcomes in the EM and in treatment settings in which bevacizumab is frequently used, i.e., early-line therapy in mCRC, mNSCLC, and mOC and second-line therapy in GBM.

## 4. Materials and Methods 

### 4.1. Approach and Participants

A questionnaire was designed to collect data on access to and usage of bevacizumab by hematologists and oncologists in the US, EU (UK, Italy, Germany, and France), and EM (Brazil, Mexico, and Turkey). Physicians were recruited via email and those who agreed to participate were administered an online survey. A brief screener survey was used to determine whether physicians were eligible to respond to the questionnaire. To participate, physicians must have been full-time hematologists, medical or clinical oncologists, pulmonary oncologists, or gynecologic oncologists, and practicing medicine for 2–35 years post-residency or fellowship in a mixture of hospital and office-based settings, with ≥50% of their professional time spent in direct patient care. Physicians must have treated ≥50 newly diagnosed or continuing cancer patients in the three months prior to responding to the survey and ≥35% of their patients must have presented with a solid tumor. Pulmonary oncologists must have treated ≥1 patient diagnosed with mNSCLC. Gynecologic oncologists must have treated ≥1 patient diagnosed with mOC or mBC, and hematologists and medical oncologists must have treated ≥1 patient for mCRC, mNSCLC, mOC, or mBC. Physicians who completed the questionnaire were monetarily compensated at rates approved by the study sponsor. The goal was to survey a total of 510 physicians: 150 in the US, 230 in the EU (UK, *n* = 20; Italy, *n* = 50; Germany, *n* = 80; and France, *n* = 80), and 130 in the EM (Brazil, *n* = 50; Mexico, *n* = 50; and Turkey, *n* = 30). 

### 4.2. Survey Details

The questionnaire focused on patients with solid tumors and included questions related to the following: type of primary malignancy, disease characteristics, line of therapy, commonly followed treatment guidelines, use of and accessibility to bevacizumab, barriers to the use of bevacizumab, and the potential use of a less-expensive (i.e., 80% of the cost of the originator) bevacizumab biosimilar if one was available. Questions pertaining to access considered all aspects of obtaining bevacizumab for a patient, including reimbursement, out-of-pocket costs to the patient, availability in practice, treatment guidelines, and regulatory approvals. Responses were provided in a variety of formats, including: numbers or percentages (e.g., what number/percentage of your patients are A, B, or C); four-, five-, six-, or seven-point scales (e.g., 1 = never and 4 = frequently); select all that apply (e.g., A through Z); select one answer only (e.g., A, B, C, D, or E); and rank top choices (e.g., top three). In certain cases, text boxes were provided for participants to further explain their answers, if necessary. Representative questions from the survey are provided in the Supplementary Methods.

### 4.3. Data

Patient load, defined as the number of patients treated in the three months prior to the survey, was analyzed by type of malignancy and line of therapy for physicians in each individual country. The percentage of physicians reporting a specific response was analyzed by market (i.e., US, EU, or EM). Results were analyzed using *t*-tests and statistically significant differences among markets or individual countries were assessed at the 95% confidence level (*p* < 0.05). 

## Supplementary Materials

The following are available online at http://www.mdpi.com/1424-8247/10/1/19/s1, Supplementary Methods: Representative questions from bevacizumab true access survey, Figure S1: Reasons for not frequently prescribing bevacizumab, by primary tumor type and line of therapy. (**A**) mCRC, (**B**) *EGFR* WT or unknown mNSCLC, (**C**) *EGFR* Mut mNSCLC, (**D**) mOC, (**E**) mBC, (**F**) GBM., Table S1: Patient load, by primary tumor type and country., and Table S2: Treatment guidelines followed by physicians, by primary tumor type.
