# Supplementary material for: Barriers to the Access of Bevacizumab in Patients with Solid Tumors and the Potential Impact of Biosimilars: A Physician Survey
Source: Pharmaceuticals (Basel). 2017 Jan 28;10(1):19. doi: 10.3390/ph10010019 (PMC5374423; doi:10.3390/ph10010019)
Supplement: Supplementary file 1 [file pharmaceuticals-10-00019-s001.pdf]

Supplementary Materials: Barriers to the Access of Bevacizumab in Patients with Solid Tumors and the Potential Impact of Biosimilars: A Physician Survey

Bradley J. Monk, Philip E. Lammers, Thomas Cartwright and Ira Jacobs

Supplementary Methods: Representative Questions from the Bevacizumab True Access Survey

Bevacizumab Use and Barriers to Treatment

What is the extent of your use of bevacizumab-based regimens across the treatment spectrum (i.e., first-line, maintenance, and second-line) for patients with mCRC, mNSCLC, mOC, mBC, and GBM?

1. Always/standard of care (>50% of cases)
2. Frequently (25%–50% of cases)
3. Not so often/occasionally for certain patients (10%–24% of cases)
4. Rarely (<10% of cases)
5. Never

What are the reasons for not frequently using bevacizumab? (Please select all that apply) (Presented for each tumor type and treatment setting where respondents cited they "Not so often/occasionally for certain patients (10%–24% of cases)," "Rarely (<10% of cases)," or "Never" prescribe bevacizumab.).

A. Patient clinical factors (i.e., age, performance status, comorbidities, contraindications, etc.)
B. I do not consider bevacizumab to be the optimal treatment option in this tumor type overall
C. Not convinced of bevacizumab's efficacy in this setting/tumor type
D. Not convinced of bevacizumab's safety in this setting/tumor type
E. Access-related issues including reimbursement, high out-of-pocket cost to the patient, availability where I practice; guidelines-regulatory authorities have not approved use in this setting
F. Other (please specify)

On an overall basis, how do you find access (i.e., reimbursement, high out-of-pocket costs to the patient, availability where you practice; and guidelines/regulatory authorities have not approved use in this setting) to bevacizumab for patients with mCRC, mNSCLC, mOC, mBC, and GBM?

Not at All Easy						Very Easy
1	2	3	4	5	6	7

Which of the following make access to bevacizumab challenging in this particular tumor type? (Please select all that apply) (Presented for each tumor type in which ease of access was considered difficult (responses ≤3). GBM, glioblastoma multiforme; mBC, metastatic breast cancer; mCRC, metastatic colorectal cancer; mNSCLC, metastatic non-squamous non–small-cell lung cancer; mOC, metastatic ovarian cancer).

A. Not reimbursed/covered by healthcare system and/or patients' private insurance
B. Not available for use in the hospital/clinic where I practice
C. Use in this tumor type/setting is not recommended by treatment guidelines/protocol I follow
D. Use in this tumor type/setting is not approved by regulatory authorities
E. High out-of-pocket treatment costs for patient

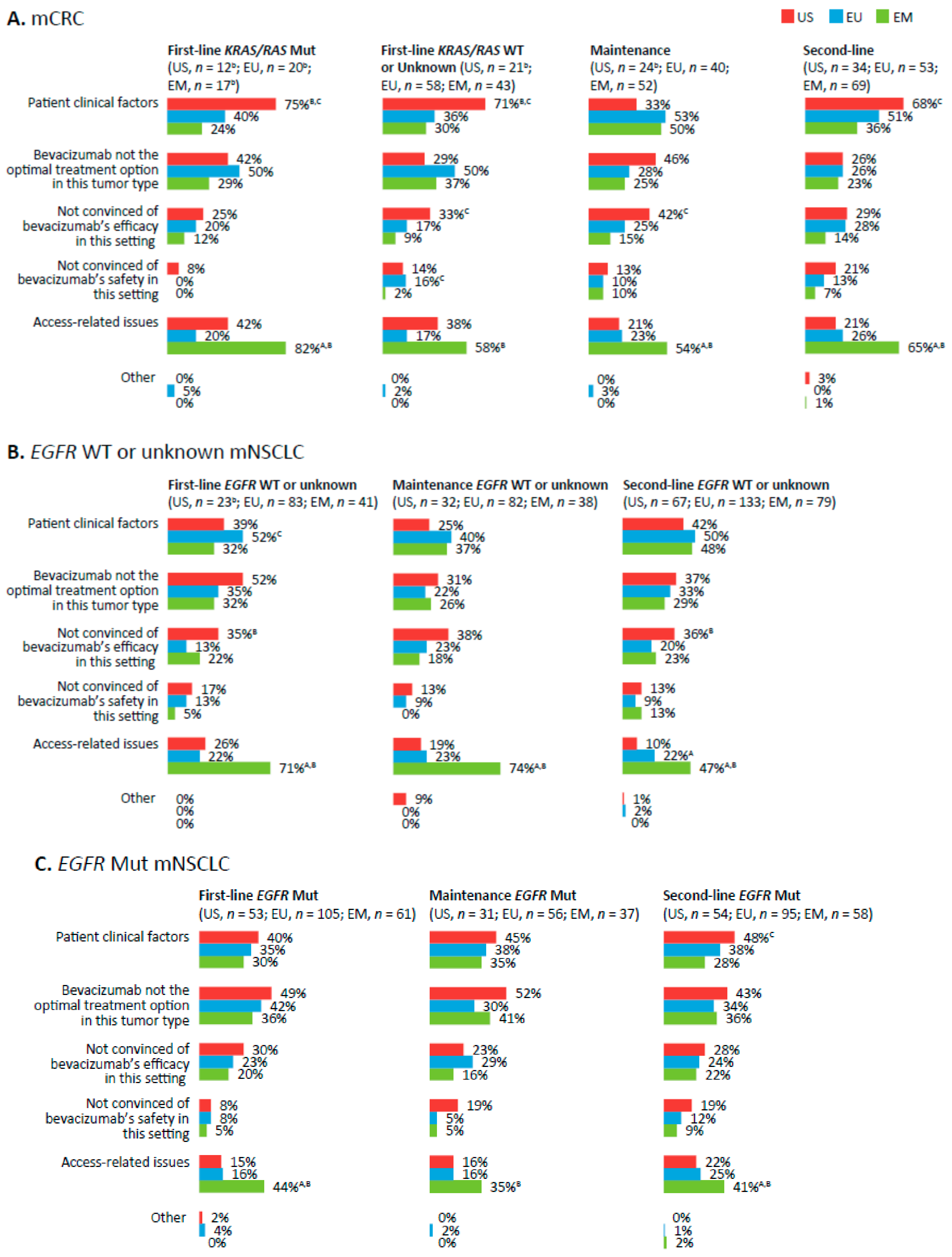
A. mCRC
US EU EM
First-line KRAS/RAS Mut (US, n = 12b; EU, n = 20b; EM, n = 17b)
First-line KRAS/RAS WT or Unknown (US, n = 21b; EU, n = 58; EM, n = 43)
Maintenance (US, n = 24b; EU, n = 40; EM, n = 52)
Second-line (US, n = 34; EU, n = 53; EM, n = 69)
Patient clinical factors
Bevacizumab not the optimal treatment option in this tumor type
Not convinced of bevacizumab's efficacy in this setting
Not convinced of bevacizumab's safety in this setting
Access-related issues
Other
B. EGFR WT or unknown mNSCLC
First-line EGFR WT or unknown (US, n = 23b; EU, n = 83; EM, n = 41)
Maintenance EGFR WT or unknown (US, n = 32; EU, n = 82; EM, n = 38)
Second-line EGFR WT or unknown (US, n = 67; EU, n = 133; EM, n = 79)
C. EGFR Mut mNSCLC
First-line EGFR Mut (US, n = 53; EU, n = 105; EM, n = 61)
Maintenance EGFR Mut (US, n = 31; EU, n = 56; EM, n = 37)
Second-line EGFR Mut (US, n = 54; EU, n = 95; EM, n = 58)

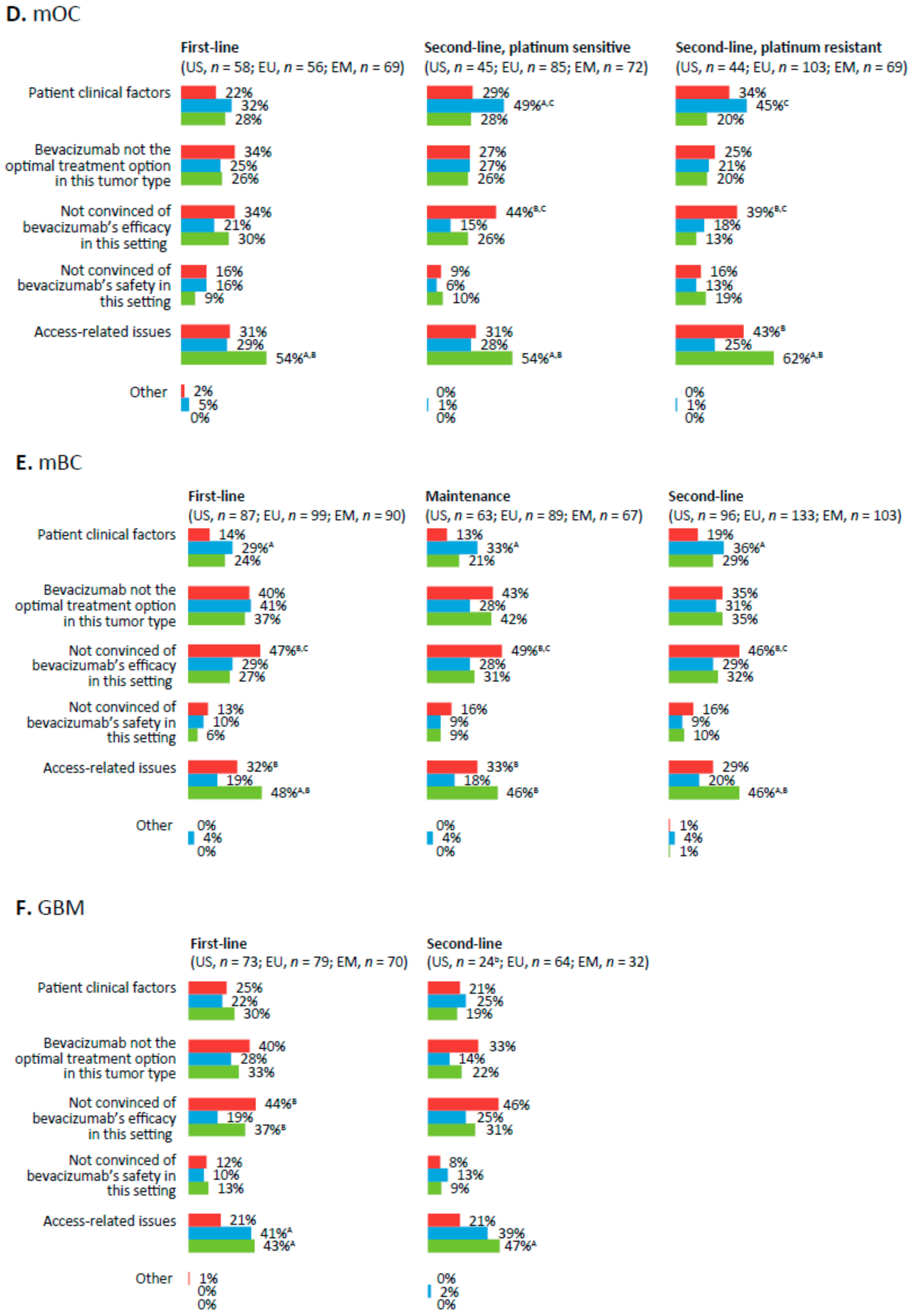

Figure S1. Reasons for not frequently prescribing bevacizumab, by primary tumor type and line of therapy[a]. **(A)** mCRC, **(B)** *EGFR* WT or unknown mNSCLC, **(C)** *EGFR* Mut mNSCLC, **(D)** mOC, **(E)** mBC, and **(F)** GBM. [A,B,C] Letters indicate a significant difference between subgroups ($p < 0.05$): A = US; B = EU; C = EM. [a] Percentages based on respondents who reported not frequently prescribing bevacizumab (a score ≥3 on a scale from 1 = always to 5 = never). [b] Small sample size; results interpreted with caution. mCRC, metastatic colorectal cancer; EGFR, epidermal growth factor receptor; WT, wild-type; mNSCLC, metastatic non-squamous non–small-cell lung cancer; Mut, mutant; mOC, metastatic ovarian cancer; mBC, metastatic breast cancer; GBM, glioblastoma; EU,

European Union (United Kingdom (UK), Italy, Germany, and France); EM, emerging markets (Brazil, Mexico, and Turkey); KRAS, Kirsten rat sarcoma viral oncogene homolog.

Table S1. Patient load, by primary tumor type and country.[a]

Primary Tumor Type	US	UK	Italy	Germany	France	Brazil	Mexico	Turkey
	No. of Patients							
	n = 150	*n* = 20[b]	*n* = 50	*n* = 80	*n* = 80	*n* = 50	*n* = 50	*n* = 30
mCRC	42[D,E,H]	54[D,E,H]	58[A,D,E,H]	28	25	45[D,E,H]	58[A,D,E,H]	18
mNSCLC	42[H]	60[A,F,H]	41[H]	43[H]	47[H]	32[H]	38[H]	15
mOC	22[H]	39[A,C,D,E,F,H]	20[H]	19[H]	20[H]	17[H]	34[A,C,D,E,F,H]	9
mBC	50[H]	66[H]	69[A,F,H]	49[H]	45[H]	63	119[A,B,C,D,E,F,H]	13
GBM	18[D,E,F,H]	14[D,E,F,H]	15[D,E,F,H]	6	6	8	13[D,E,F,H]	7

[A,B,C,D,E,F,G,H] Letters indicate a significant difference between subgroups ($p < 0.05$): US = A; UK = B; Italy = C; Germany = D; France = E; Brazil = F; Mexico = G; Turkey = H. [a] Values represent the number of patients treated by physicians over the past three months. Sample size includes respondents who reported treating patients who had each primary tumor type. [b] Small sample size; results interpreted with caution. mCRC, metastatic colorectal cancer; mNSCLC, metastatic non-squamous non–small-cell lung cancer; mOC, metastatic ovarian cancer; mBC, metastatic breast cancer; GBM, glioblastoma.

Table S2. Treatment guidelines followed by physicians, by primary tumor type.[a]

Guideline, by Primary Tumor Type	US	EU	EM
	Percentage (%) of Respondents		
mCRC	*n* = 150	*n* = 150	*n* = 130
NCCN	79[B]	43	82[B]
ASCO	33	38	52[A,B]
Hospital guidelines	16	24[C]	13
ESMO	0	52	48
National guidelines/Ministry of Health	0	23[C]	9
None[b]	7	5	0
mNSCLC	*n* = 150	*n* = 190	*n* = 130
NCCN	79[B]	38	75[B]
ASCO	35	47[A]	56[A]
Hospital guidelines	11	23[A,C]	10
ESMO	0	51	40
National guidelines/Ministry of Health	0	23	18
None[b]	8	5	0
mOC	*n* = 150	*n* = 190	*n* = 130
NCCN	75[B]	32	79[B]
ASCO	34	37	48[A,B]
Hospital guidelines	9	21[A,C]	10
ESMO	0	46	41
National guidelines/Ministry of Health	0	24[C]	9
None[b]	9	5	0
mBC	*n* = 150	*n* = 190	*n* = 130
NCCN	77[B]	32	78[B]
ASCO	32	34	55[A,B]
Hospital guidelines	11	23[A,C]	8
ESMO	0	42	52
National guidelines/Ministry of Health	0	23[C]	12
None[b]	8[B]	2	0
GBM	*n* = 142	*n* = 127	*n* = 123

NCCN	76[B]	37	73[B]
ASCO	28	30	46[A,B]
Hospital guidelines	11[C]	21[A,C]	3
ESMO	0	42	42
National guidelines/Ministry of Health	0	22[C]	7
None[b]	11[C]	9[C]	1

[A,B,C] Letters indicate a significant difference between subgroups ($p < 0.05$): A = US; B = EU; C = EM. [a] Percentages based on respondents who reported treating patients who had each primary tumor type. [b] A response of "None" indicates that no guidelines/protocol were followed. EU, European Union (United Kingdom (UK), Italy, Germany, and France); EM, emerging markets (Brazil, Mexico, and Turkey); mCRC, metastatic colorectal cancer; NCCN, National Comprehensive Cancer Network; ASCO, American Society of Clinical Oncology; ESMO, European Society for Medical Oncology; mNSCLC, metastatic non-squamous non–small-cell lung cancer; mOC, metastatic ovarian cancer; mBC, metastatic breast cancer; GBM, glioblastoma.
